# Phage-Antibiotic Synergy (PAS): β-Lactam and Quinolone Antibiotics Stimulate Virulent Phage Growth

**DOI:** 10.1371/journal.pone.0000799

**Published:** 2007-08-29

**Authors:** André M. Comeau, Françoise Tétart, Sabrina N. Trojet, Marie-Françoise Prère, H.M. Krisch

**Affiliations:** 1 Laboratoire de Microbiologie et Génétique Moléculaires, Centre National de la Recherche Scientifique, Université Paul Sabatier-Toulouse, Toulouse, France; 2 Laboratoire de Bactériologie, Institut Fédératif de Biologie, Centre Hospitalier Universitaire de Toulouse, Toulouse, France; The Research Institute for Children, United States of America

## Abstract

Although the multiplication of bacteriophages (phages) has a substantial impact on the biosphere, comparatively little is known about how the external environment affects phage production. Here we report that sub-lethal concentrations of certain antibiotics can substantially stimulate the host bacterial cell's production of some virulent phage. For example, a low dosage of cefotaxime, a cephalosporin, increased an uropathogenic *Escherichia coli* strain's production of the phage ΦMFP by more than 7-fold. We name this phenomenon Phage-Antibiotic Synergy (PAS). A related effect was observed in diverse host-phage systems, including the T4-like phages, with β-lactam and quinolone antibiotics, as well as mitomycin C. A common characteristic of these antibiotics is that they inhibit bacterial cell division and trigger the SOS system. We therefore examined the PAS effect within the context of the bacterial SOS and filamentation responses. We found that the PAS effect appears SOS-independent and is primarily a consequence of cellular filamentation; it is mimicked by cells that constitutively filament. The fact that completely unrelated phages manifest this phenomenon suggests that it confers an important and general advantage to the phages.

## Introduction

The phage-infected bacterial cell undergoes a complex developmental program that is largely defined by the phage genome. Although this developmental cycle is generally well understood for phages such as T4 or λ [1], [2], little is known about how the external environment influences it. What is well known, however, is that many classes of antibiotics inhibit bacterial cell division [3], [4] and can thus, at sub-lethal concentrations, increase the biomass and hence the biosynthetic capacity of bacterial cells. A fortuitous observation has allowed us to demonstrate that virulent phages have evolved the capacity to take advantage of this altered host cell state to increase their own production. A second consequence of such drugs is to accelerate the lysis of the infected cells, hence allowing the phages to spread more rapidly. We discuss the observations that lead to the discovery of this phenomenon, its characteristics, and the role that this phage-antibiotic synergy could play in the natural environment.

## Results and Discussion

**Figure 1:**
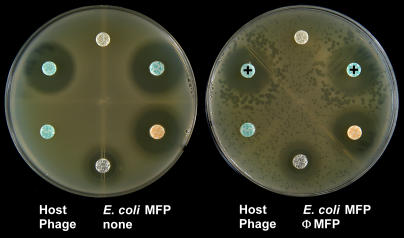
The PAS effect of phage ΦMFP on *E. coli* MFP on Luria-Bertani agar plates. Only disks containing the β-lactam antibiotics aztreonam and cefixime (indicated by “+” symbols) produced large phage plaques in their proximity. Gentamicin and tetracycline gave no PAS effect. This host strain was resistant to both amoxicillin and trimethoprim/sulfamethoxazole. Note the absence of phages on the left-hand control plate indicating the lack of prophage induction.

To determine the antibiotic sensitivity of the bacterium causing a urinary tract infection in a hospitalized child, a Petri plate was inoculated with a dilution of the patient's urine and a series of antibiotic disks were placed on the surface of the agar. As suspected, the urine was contaminated with an uropathogenic *Escherichia coli* strain, but also with phages that infected it. Remarkably, with some of the antibiotics, the phage plaques were significantly larger in zones circumscribing the disks where there was a sub-lethal concentration of the drug. This effect was observed with disks containing the β-lactam antibiotics (aztreonam, a monobactam, and cefixime, a cephalosporin), but not with antibiotics of other classes (tetracycline and gentamicin). Thus, a sub-lethal dosage of β-lactam antibiotics appeared to stimulate phage growth in this host-phage system. To further characterize this curious phenomenon, both a single a colony of the uropathogenic *E. coli* isolate (MFP) and a single phage plaque (ΦMFP) were isolated from the urine sample. The synergistic effect was highly reproducible with these purified isolates (Figure 1) and was not the result of induction of lysogens (no appearance of phage on control plate)–we called this effect PAS (**P**hage **A**ntibiotic **S**ynergy). Other cephalosporins (cefotaxime, ceftriaxone, and ceftazidime) produced a similar effect on ΦMFP growth in the *E. coli* MFP strain. Since this host strain is resistant to many types of penicillins (e.g. piperacillin, ticarcillin and amoxicillin), these drugs in the β-lactam family could not be tested.

Electron microscopy of the phage ΦMFP revealed morphology typical of a siphovirus [5]: a long, flexible, non-contractile tail structure and an isometric, icosahedral head of ∼60 nm (data not shown). DNA sequencing of 25 random ∼500 bp segments of the ΦMFP genome indicated that it is related to the *Salmonella typhimurium* phage MB78 [6] (data not shown).

**Figure 2:**
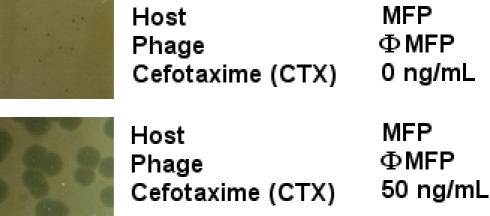
Plaque sizes of phage ΦMFP on *E. coli* MFP with and without 50 ng/mL of cefotaxime (CTX) in Luria agar plates.

Since the PAS effect was only detectable in the zone adjacent to the disk where the drug was not completely inhibiting bacterial growth, we determined the drug concentration in Luria-Bertani (LB) agar that gave the optimal PAS effect. These plates were overlaid with soft LB agar containing the MFP indicator strain, a fixed quantity of the ΦMFP phage and various concentrations of the cephalosporin cefotaxime. Up to a concentration of 50 ng/mL, the PAS response (as assayed by plaque diameter) increased with the dose. As shown in Figure 2, this phage made quite small plaques with no cefotaxime and remarkably large ones with the optimal concentration, 50 ng/mL. Concentrations of cefotaxime above 100 ng/mL inhibited the growth of the MFP bacterial strain to a level that made the analysis phage plaque size impossible.

**Figure 3:**
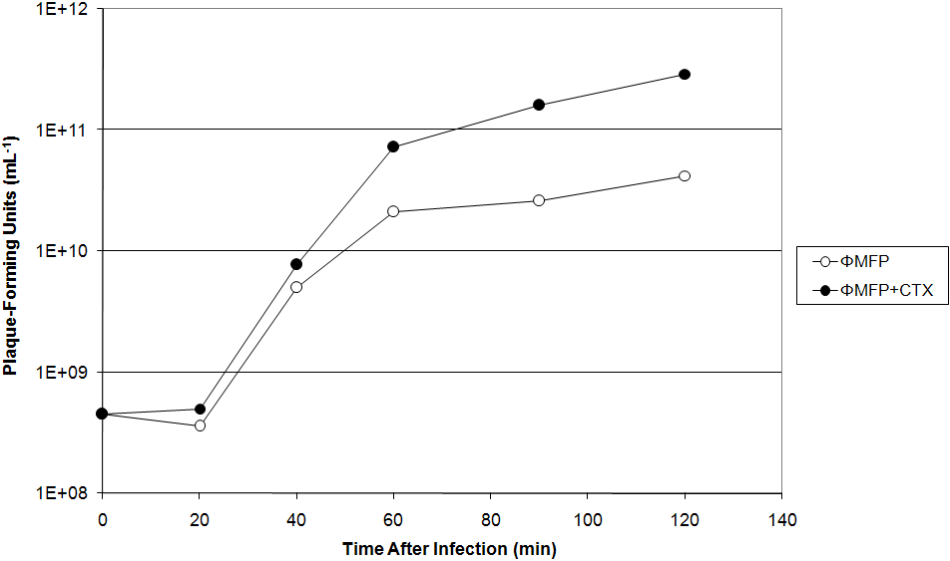
Increase in phage titer in the presence of the cephalosporin cefotaxime (CTX). *E. coli* strain MFP was infected with phage ΦMFP in Luria liquid medium supplemented at the time of infection with 20 ng/mL of cefotaxime, or left untreated. The multiplicity of infection was ∼5. Chloroform was added at various times after infection to lyse the infected cells.

Plaque size is largely determined by two properties of the phage infected cell: the phage burst size (the number of virions produced by each infected cell) and the time it takes the phage to lyse an infected cell. To determine if the cefotaxime-mediated increase in plaque size was due directly to an increase in phage burst size, we compared phage ΦMFP production when the MFP host cells were grown in a liquid culture either with or without 20 ng/mL of cefotaxime. The data in Figure 3 show that total phage production in a single cycle of growth was greater in the presence of the cephalosporin. This difference was detectable throughout the latent period and was about 7-fold when the two cultures lysed. However, the lysis of the antibiotic-treated infected culture was also clearly affected, occurring more rapidly in the presence of the drug (data not shown). It should be recalled that some mutant phages lyse more rapidly and also make significantly larger plaques than their wild type ancestor [7].

**Figure 4:**
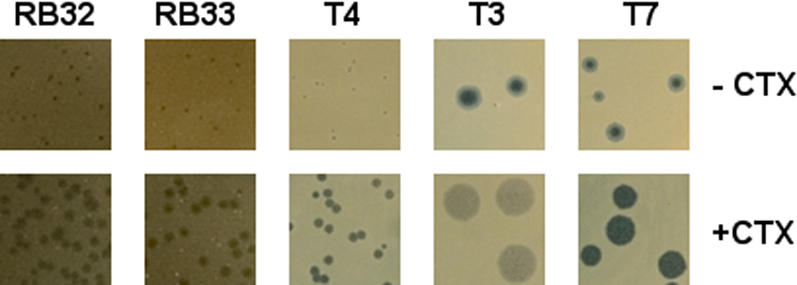
Plaque sizes of various coliphages with and without cefotaxime (CTX) in Luria agar plates. Phages RB32 and RB33 were grown on *E. coli* strain MFP (50 ng/mL CTX) at 37°C; and T4, T3 and T7 were grown on *E. coli* strain AS19 (30 ng/mL CTX) at 25°C. All plaques were photographed at identical magnifications.

A search of the phage literature revealed that a possibly related observation had been made in the classic T4 phage system. In 1969, Yamagami and Endo [8] observed that UV irradiation, mitomycin C and a variety of other chemical treatments that cause *E. coli* B to form filaments resulted in T4 plaques that were larger than normal. To determine if the PAS phenomenon also occurred in the T4-type phages, we identified two phages, RB32 and RB33 [9], in the Toulouse collection that grow well on *E. coli* MFP. These phages have a contractile tail and are thus classified as myoviruses [5], a phage family phylogenetically distinct from the siphoviruses, such as ΦMFP. Both T4-type phages gave a clear PAS response, in other words, much larger plaques when plated on the host MFP in the presence of cefotaxime (Figure 4). A large set of T4-like phages from the Toulouse collection were subsequently tested on various standard laboratory *E. coli* strains and most, but not all (e.g.: RB43), of these gave a positive PAS response on host strains such as C600, P400, MC1061, S/6 and AS19. The strain AS19, an antibiotic permeability mutant [10] of *E. coli* B, was chosen for further studies of the PAS effect because this host gave the most dramatic increase in plaque size for the greatest number of T4-type phages tested. AS19 is very sensitive to most β-lactam antibiotics, so additional drugs in this family could be tested for PAS and many of these gave a positive PAS response (data not shown). For example, the drugs ticarcillin, piperacillin, ampicillin and, surprisingly, the structurally unrelated quinolone nalidixic acid all increased T4 phage plaque size significantly. Furthermore, the PAS response is not restricted to phage infections in *E. coli*; we could demonstrate the phenomenon in an infection of the phylogenetically distant host *Yersinia pseudotuberculosis* in an infection with the T4-type *Yersinia* phage PST (data not shown). The generality of the PAS phenomenon was further extended by showing that phages T3 and T7, belonging to the phylogenetically distant podoviruses [5] (the short-tailed family of phage), also gave an impressive PAS response on the host AS19 (Figure 4).

To further examine the PAS effect in the T4-like phages, we collected the soft agar containing AS19 host lawns from plates containing a fixed, non-confluent, quantity of T4 plaques that had been incubated the same amount of time either with or without the antibiotic. The titers of these plate lysis stocks were then compared. Phage T4 indeed showed an ∼11-fold greater titer when grown in the presence of 30 ng/mL of cefotaxime, and an ∼9-fold increase in the presence of 200 ng/mL of mitomycin C. To directly examine the kinetics of the T4 phage production, we performed single burst experiments in liquid cultures of AS19 host cells that were pre-treated with cefotaxime and then infected with either T4 or two other T4-type phages, RB33 and RB49 [9]. In such single cycle growth experiments, there was actually a slight reduction in phage production, but this small effect was coupled to a very significant reduction in the time to lyse the infected culture (only 75–90 min compared to the normal 2 h). This result provoked us to examine the effect of the antibiotics on T4 gene *t* mutants which lack the holin gene that is primarily responsible for the timing of host cell lysis [11]. Such phage mutants have much delayed lysis, but the presence of the antibiotics largely suppressed this phenotype (data not shown), consistent with the drugs having a postulated effect of accelerating the time of lysis.

The PAS response appears to be explained by a simple mechanism. There is a striking effect on cell morphology when sensitive strains of *E. coli* grow in the presence of even very low levels of β-lactam antibiotics. The mode of action of these antibiotics is to block cell division [3] and, even with the sub-lethal dosages that we employed, the cells divide poorly and form very long filaments that eventually fatten somewhat. Furthermore, we have verified by light microscopy that the cells filament in the circular zone surrounding the antibiotic disks where the PAS response occurs. Significantly, the chemically unrelated quinolone antibiotic, nalidixic acid, which stimulates phage growth at levels similar to the β-lactams also acts by inhibiting bacterial cell division, but by a different mechanism [12]; as do mitomycin C [13] and UV irradiation [14]. The SOS response in *E. coli* has long been known to cause an inhibition of bacterial cell division in response to DNA damage. Interestingly in the context of our results, it has recently been demonstrated that the inactivation of the *ftsI* gene product, the penicillin binding protein III, by β-lactam antibiotics also induces the expression of the SOS system via a two-component signal transduction system [15]. Among the numerous genes in the SOS regulon is the *sulA* (*sfiA*) gene that encodes an inhibitor of cell division. Thus, the bacterial SOS response to β-lactam antibiotics results in filamentation that allows the bacteria to reduce the lethal effects of the drugs [15].

**Figure 5:**
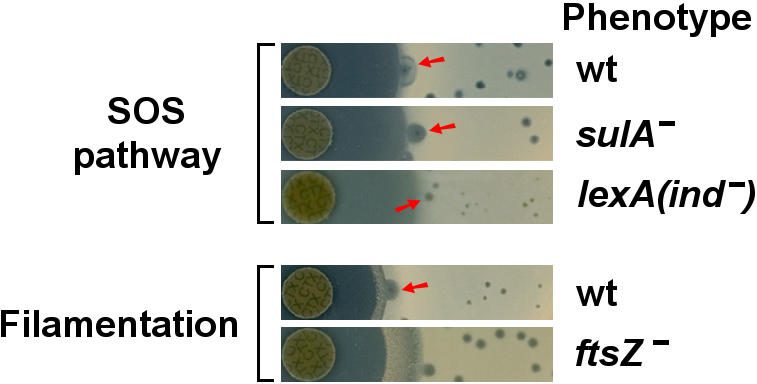
The PAS effect of phage T4 on various *E. coli* SOS and filamentation mutant strains. T4 was grown on *E. coli sulA*-inactivated and *lexA* non-inducible mutant strains (defective SOS systems), as well as an *ftsZ*-inactivated mutant strain (non-antibiotic induced filamentation), in the presence of disks of cefotaxime (CTX). Isogenic wild-type strains (wt) are also included and representative plaques demonstrating the PAS effect are indicated by red arrows. All plates were photographed at identical magnifications.

Obvious questions arise concerning a role of the bacterial SOS response, and filamentation in general, in the PAS phenomenon. To address these questions we employed a series of *E. coli* mutant hosts, that were either defective in the SOS pathway or had an altered filamentation response (constitutive filamentation), and determined if they gave the PAS effect or not (Fig. 5). These results indicate that the PAS effect is SOS pathway-independent as larger plaques around the antibiotic halos were clearly present in the *sulA* mutant, as well as in a *lexA* mutant which cannot de-repress the SOS pathway genes (and hence should have no SOS response). A *recA* mutant, defective for a major component of the SOS system, showed a positive, but slightly dimished, PAS response (data not shown). Thus, the SOS system does not seem to play a primary role in the PAS response, it could nevertheless still play some minor supporting role. However, the primarily SOS-independent nature of the PAS effect may not be surprising given that filamentation can still be induced by antibiotics in an SOS-independent manner [16]. Turning to filamentation itself–if the PAS effect is primarily due to the physiological changes in filamenting cells, a mutant *E. coli* strain that is able to filament without the activity of antibiotics should have a similar response. That is exactly what is observed (Fig. 5) in an *ftsZ* mutant which filaments due to its defective cell division apparatus. Phage plaques on this mutant are uniformly bigger than the isogenic control strain throughout the host lawn with only a very modest increase in size near the zone of antibiotic activity.

The phenotype of filamentation therefore seems to play a significant role in the PAS effect, probably through multiple pathways. The altered physiological state of filamenting cells obviously permits much faster phage assembly, possibly by making larger/altered pools of precursors available and/or remedying certain rate-limiting steps in assembly. We suggest that the antibiotics also accelerate the timing of cell lysis given that filamentation induces perturbations in the peptidoglycan layer and this probably causes a greater sensitivity to the action of phage lysis genes (e.g. lysozymes, holins). The interplay of these two effects, faster lysis and increased rate of phage production, cause the PAS effect and it is not surprising that different phage-host systems, with their different developmental programs and molecular mechanisms of lysis, respond slightly differently. For example, there is a significant increase in production in the siphophage MFP-*E. coli* MFP system, whereas T4-infected *E. coli* AS19 does not increase phage production, but does significantly reduce the time of the infectious cycle in these T4-like phages. As a consequence, the phages are produced more rapidly and hence can diffuse further than normally, which causes an increase in plaque size on solid media.

It is self-evident that the significant increases in phage production reported here could be useful in certain biomedical (e.g. phage therapy) or biotechnological applications. A more fundamental question is whether the PAS response is simply a useful curiosity, or a reflection of a previously unappreciated aspect of the phage life-cycles, the ability to successfully adapt to a “sub-optimal” environment for bacterial growth. The presence in the environment of low levels of antibiotics that interfere with cell wall synthesis represents a major threat to bacteria and it was to be expected that they would develop strategies to minimize the consequences of such agents. The SOS system induction of filamentation can reduce the danger posed by such compounds [15] and is thus evolutionarily advantageous. Phage of both the temperate (induction of lysogens [17]) and virulent (PAS-enabled) types have adapted to take advantage of the altered physiology of such stressed cells and can propagate adventitiously in them, producing more progeny than they would in “healthier” situations. This makes sense from an evolutionary perspective since the host cells are likely to die anyway and getting a last, quick burst of phage out of them before they do so is to the advantage of the phage. From an ecological perspective, this seemingly bizarre situation can be viewed as a mutualism between the fungal or bacterial cells producing the antibiotics and PAS-enabled phages to more efficiently compete with antibiotic- and phage-sensitive bacterial cells. This synergistic interaction between phages and antibiotic-producing cells could play a role in determining population balances in the microfauna of biofilms, for example. Clearly, more investigation will be required to assess the impact that PAS has on the microbial ecosystem. With respect to the clinical setting, there is perhaps a lesson to be learned here from nature–if your objective is to kill bacteria, then giving them a tad of β-lactam antibiotics combined with a dash of phage may be better at accomplishing this task than either treatment individually.

## Materials and Methods

### Phages and bacteria

All phages and bacteria were from the Toulouse Laboratory collection of T4-like phages and their hosts, except for the following: the phage-host system MFP was isolated from a urine sample of an infected pediatrics patient using streak plating and standard phage techniques [1]; phages T3 and T7 were kindly provided by Ian Molineaux (University of Texas); *E. coli* SOS and filamentation mutants were kindly provided by our LMGM colleagues, Kaymeuang Cam, François Cornet and David Lane. The SOS mutants employed were *sulA*-inactivated (Coli Genetic Stock Center [CGSC] strain AB1157 with *sulA::Tn5*), *recA*-inactivated (CGSC strain AB2463) or *lexA* non-inducible (CGSC strain AB2494) compared to the isogenic wild-type strain (CGSC strain AB1157). The filamentation mutant employed was *ftsZ*-inactivated (strain SK686 = *ftsZ84* in MG1655 Δ*lac*) compared to the isogenic wild-type strain (strain SK683 = MG1655 Δ*lac*).

### Antibiotics and phage production assays

Antibiotic disks for solid assays were obtained from Bio-Rad; powdered drugs for liquid assays and soft agar overlays were obtained from Sigma and diluted to the desired concentrations in sterile water. Liquid one-step growth curves, agar overlays and solid plate stocks were performed as described previously [1], with the addition of the appropriate antibiotic types/forms (disks or solutions) to the appropriate media.
